# Protocol for Development of Guidelines for Rapid Restoration of Readiness

**DOI:** 10.1227/neuprac.0000000000000274

**Published:** 2026-08-13

**Authors:** Simon Oczkowski, Thomas Bayuk, Bradley A. Dengler, Michael McCrea, Sameer Sharif, Katie Stout, Gregory W. J. Hawryluk, Halinder S. Mangat, Jamshid Ghajar

**Affiliations:** 1 Division of Critical Care, Department of Medicine, McMaster University, Hamilton, Ontario, Canada;; 2 Department of Health Research Methods, Evidence, and Impact, McMaster University, Hamilton, Ontario, Canada;; 3 Guidelines in Intensive Care, Development, and Evaluation (GUIDE) Group, Hamilton, Ontario, Canada;; 4 Department of Neurology, Uniformed Services University of the Health Sciences, Bethesda, Maryland, USA;; 5 Department of Surgery, Uniformed Services University of the Health Sciences (USU), Bethesda, Maryland, USA;; 6 Department of Neurosurgery, Walter Reed National Military Medical Center, Bethesda, Maryland, USA;; 7 Military Traumatic Brain Injury Initiative (MTBI^2^), Bethesda, Maryland, USA;; 8 Department of Neurology, Uniformed Services University of the Health Sciences (USU), Bethesda, Maryland, USA;; 9 Department of Neurosurgery, Medical College of Wisconsin, Milwaukee, Wisconsin, USA;; 10 Division of Emergency Medicine, Department of Medicine, McMaster University, Hamilton, Ontario, Canada;; 11 Traumatic Brain Injury Center of Excellence, Defense Health Agency, Silver Spring, Maryland, USA;; 12 Cleveland Clinic Lerner College of Medicine, Case Western Reserve University School of Medicine, Cleveland, Ohio, USA;; 13 Brain Trauma Foundation, Menlo Park, California, USA;; 14 Kansas University Medical Center Research Institute, Kansas City, Kansas, USA

**Keywords:** Attention, Concussion, Sleep, Rehabilitation, Supplements, Stimulants, Sport

## Abstract

Combat and sport require individuals to be ready for successful completion of the task at hand. Cognitive readiness and attention may be impaired for many reasons, including concussion or subconcussive injury, insufficient sleep, fatigue, or any other disruption or deviation of baseline physiology. While rapid readiness screening may identify individuals who are not “ready” for a given task, it is also important to find interventions which can rapidly return an individual to a state of readiness. Although general health practices such as nutrition, physical conditioning, and adequate rest support overall performance, targeted interventions—focused on oculo-vestibular, verbal-cognitive function, and motor control—may accelerate return to readiness. Additional modalities such as structured sleep optimization protocols, controlled stimulant use, and specific supplements may also contribute to rapid restoration. This paper describes an evidence-based framework for developing guidelines that identify, evaluate, and implement rapid readiness restoration strategies across diverse preclinical settings, including military far-forward environments, sports sidelines, and garrison or training contexts.

**ABBREVIATIONS:**
COI: conflict of interest
DoW: Department of War
GRADE: Grading of Recommendations Assessment, Development, and Evaluation
PICO: population, intervention, comparator, outcome
RRS: rapid readiness screen.

In many settings, including military and athletics, optimizing performance is critical within a time-constrained and resource-constrained environment. There is an important gap between returning to the field and removal for detailed assessment^1^—a need for a rapid, objective screening tool to assess baseline cognitive performance and detect changes after potential neurological or physiological impairment, along with simple, practical interventions that can be implemented in those settings to support safe return to military duty or sport activity, with minimal training or resource requirements.

This aligns with a central goal of the Department of War (DoW) to strengthen the cognitive and physical performance of Warfighters to ensure they are prepared for deployment and able to safely and effectively return to duty after injury. Through the Warfighter Brain Health Initiative, the DoW advances research focused on assessing, protecting, and improving brain function in response to the intense cognitive and physical demands of military service, including sleep deprivation, concussive and subconcussive blast exposure, environmental hazards, and psychological stress.^2^ Sustaining optimal cognitive readiness is critical to operational capability and mission success.

In this project, we define cognitive readiness as “an optimal brain state that enables precise coordination of sensory inputs and motor outputs appropriate to the task at hand.” However, current measures of readiness do not adequately assess a Warfighter's ability to sustain performance in the demanding and dynamic environments in which they operate. The 2017 report, *Military Cognitive Performance and Readiness Assessment Initiative*, underscored the absence of objective tools capable of predicting performance, assessing injury risk, or guiding restoration.^3^ Multimodal indicators—including speech patterns, eye movement metrics, and postural stability—are critical for assessing cognitive readiness and attention and can also inform targeted restoration strategies.

The Brain Trauma Foundation recently developed guidelines for a Rapid Readiness Screen (RRS) to establish an evidence-based framework for multimodal, rapid assessments of readiness.^4^ Structured with reference to the Glasgow Coma Scale, the RRS organizes assessment domains into eye, verbal, and motor domains. An expert panel further developed an adaptive algorithm designed for a broad range of operational environments—including far-forward military settings, sports sidelines, garrison units, and clinical facilities—providing a scalable threshold-based method to assess operational readiness.

The DoW recognizes the urgent need for evidence-based restorative interventions—particularly for concussion, subconcussive blast exposure, and sleep-related fatigue—to return Warfighters to duty and maintain mission performance. With the introduction of RRS thresholds tailored to specific operational tasks, the ability to rapidly restore cognitive and attentional function becomes essential, especially in nonclinical environments. These Rapid Restoration of Readiness guidelines are designed to meet that need, enabling timely restoration of readiness before resource-intensive clinical evaluation pathways are available. While immediately applicable to military and sport, they may be of value in other time-limited and resource-limited settings where optimal cognitive readiness is essential.

## METHODS

### Working Group Membership

A steering committee was convened by the Brain Trauma Foundation leadership to provide project governance, including selection of project members. Project panel members were chosen for subject matter expertise on rehabilitation and optimization in relevant domains (eye, verbal, motor, sleep) and contexts (military, sports). Panel members were divided into working groups, each assigned to one guideline question. Methodology support throughout the guideline is provided by the Guidelines in Intensive Care Medicine, Development, and Evaluation Group, which brings expertise in the use of Grading of Recommendations Assessment, Development, and Evaluation (GRADE) methodology for guideline development.^5^

### Conflict of Interest Identification and Management

A structured conflict of interest (COI) policy will be followed, with all panelists required to disclose potential COIs of any nature, either financial (any relationship to an entity related to a guideline question) or intellectual (defined as being senior/first author or grant holder on any papers included in the guideline). Panelists with potential intellectual COIs may participate in reviewing and interpreting the evidence but will be recused from participating in discussion or writing of recommendations or consensus statements on any topic where the COI is relevant.

### Question Selection

General interventions to promote readiness in individuals without specific impairments—related to diet, exercise, and healthy sleep—were considered “in scope” for the guideline but would consist of summaries of current recommendations from the US military rather than new recommendations. Instead, the guideline would focus its resources on restoring and optimizing function in individuals with potential impairment, based upon the RRS. The steering committee identified interventions that might enhance readiness related to each section of the RRS (eye, verbal, and motor components). Three additional categories of interventions (structured sleep, stimulants, and supplements) were also added after discussion with subject matter experts on the steering committee. These interventions were structured by the methodology team in the population, intervention, comparator, outcome (PICO) format, comprising a total of 6 questions (Table 1).

**TABLE 1.** PICO Questions and Inclusion/Exclusion Criteria

PICO topic	Population	Interventions	Comparators	Study designs	Exclusion
Eye	Individuals age 14-65, baseline healthy and in military service/athletics AND Impaired readiness; with potentially impaired cognition/attention or function due to one or more of mild traumatic brain injury or potential concussion/subconcussive head injury or sleep deprivation or baseline function AND Within ∼4 wk of event AND In a garrison/austere nonhospital environment	Vision therapy, orthoptics, prism, concussion physical therapy (visual), vestibular physiotherapy, oculomotor rehab, neurooptometric rehabilitation; smooth pursuits, saccades, accommodation convergence, vestibulo-ocular reflex, pencil pushup rehab, canalith repositioning maneuvers	Placebo or usual care	Randomized controlled trials (parallel-arm, individual patient crossover, or cluster trials)	Only published as abstract non-English; published before 2000
Verbal		Cognitive rehabilitation, cognitive training, mindfulness (grounding techniques, nature techniques, resiliency training), biofeedback, voice biomarker, working memory training, single session psychotherapy			
Motor		Static balance; dynamic balance; postural control; quiet stance; vestibular rehabilitation; sensory-motor integration training, coordination training, gaze stability training; perturbation training; lower extremity strength training, cervical manual therapy			
Sleep		Sleep interventions (naps, sleep duration, quality, interventions to enhance sleep—neurostimulation, melatonin, light therapy, acoustic simulation, binaural beats) (note: not “rest” or “inactivity” after injury)			
Stimulants		Prescription or nonprescription oral or patch stimulants (including caffeine, nicotine, amphetamines, modafinil, psychedelics, ketamine)			
Supplements		Any dietary supplement other than stimulants (e.g., taurine, vitamin B12, creatine, exogenous ketones)			

PICO, population, intervention, comparator, outcome.

### Definition and Classification of Outcomes

The steering committee prioritized outcomes as “critical,” “important,” or “unimportant” for decision making in accordance with GRADE.^6^ Thresholds for trivial, small, moderate, and large effects were set to provide a contextual approach to determining imprecision of results^7^ (Table 2). Three outcomes were considered “critical” for decision making:

1. Time to return to combat/sports: Because the steering committee recognized that interventions in the guideline would be expected to have effects on different time scales from days to weeks (rehabilitation, supplements), hours to days (sleep), and hours to minutes (stimulants), separate effect size thresholds were set for each.
2. Measures of performance in the field: These would include any measurements of actual performance under real-world conditions.
3. Injury rate: Injury and reinjury are major determinants of an individual's ability to perform over long periods of time.

**TABLE 2.** Outcome Ratings and Effects Size

	Trivial effect	Small effect	Moderate effect	Large effect
Critical outcomes				
Time to return combat/sport [stimulants]	<0.25 d	0.25-<1 d	1-2 d	More than 2 d
Time to return combat/sport [sleep]	<0.5 d	0.5-<1.5 d	1.5-2.5 d	More than 2.5 d
Time to return combat/sport [rehabilitation]	<1 d	1-<2 d	2-5 d	More than 5 d
Injury rate	<1%	1%-<2%	2%-5%	More than 5%
Measures of performance	<0.2 SD	0.2-<0.5 SD	0.5-0.8 SD	More than 0.8 SD
Important outcomes				
Measures of readiness/attention/cognition	<0.2 SD	0.2-<0.5 SD	0.5-0.8 SD	More than 0.8 SD
Concussion/head injury symptoms	<0.2 SD	0.2-<0.5 SD	0.5-0.8 SD	More than 0.8 SD
Requirement for long-term follow-up and/or long-term disability	<1%	1%-<2%	2%-5%	More than 5%

Three outcomes were considered “important” but not critical for decision making. Two of these were surrogate outcomes for suggesting an individual's readiness to return at a given level of function:

1. Measures of readiness/attention/cognition: These would include test scores and other surrogate measurements of readiness and attention.
2. Presence of symptoms, particularly those related to Vestibular Ocular Motor Screening (dizziness, nausea, fatigue, brain fog, headache).^8^

A third “important” outcome was added: to identify whether the interventions may have detrimental long-term effects, such as disability or requirement for long-term follow-up; the panel recognized that while short-term restoration of readiness is the priority, it would be important to know if there are potential trade-offs between short-term readiness and long-term complications.

### Literature Review

Each PICO question will be informed using a systematic literature review, following Preferred Reporting Items for Systematic Reviews and Meta-Analyses reporting guidelines.^9^ A medical librarian will use key terms and previously published systematic reviews on each PICO question to develop electronic database searches of the most common medical databases—MEDLINE, Embase, and Cochrane Central—for studies from 2000 to present, recognizing that older publications may not reflect current technology or contemporary practice. Each search strategy will undergo peer review before final retrieval of search results and will be published as supplemental material in the final guideline paper.^10^ Supplemental literature searches for additional papers will be conducted using Elicit AI and notated in the Preferred Reporting Items for Systematic Reviews and Meta-Analyses diagrams.

### Inclusion Process for Articles

Data base search results will be uploaded for screening in COVIDENCE (Covidence systematic review software 2025, Veritas Health Innovation. Available at https://www.covidence.org). Table 1 presents detailed inclusion/exclusion criteria for each PICO. Across all PICOs, we will include studies with >50% patients aged 16 to 45, including previously healthy volunteers, athletes, or military-aged personnel, under acute stress or testing, with potential impairment of cognition/attention/function. If impairment is due to possible head injury, we would include studies of participants in the early postinjury period, within 4 weeks of an inciting injury. This population would include patients with early mild traumatic brain injury, concussion, subconcussive injury, sleep deprivation, or acute stress from training in high-level athletics or testing. Indirect evidence of related populations will be considered for inclusion where direct evidence is lacking and will be downgraded for indirectness as per GRADE standards.^11^ Interventions that could be feasible in an austere or garrison environment with minimal equipment will be included. Eligible study types will include randomized controlled trials of any design, including parallel-arm, cluster trials, and crossover trials. We will exclude trials with insufficient evidence to allow for accurate data extraction and critical appraisal, such as references published in abstract form only or those not published in English. Two reviewers will screen all retrieved references independently as titles and abstracts and subsequently as full-text reviews. Retrieved references will be screened independently for inclusion by 2 reviewers as titles and abstracts and at a secondary full-text review stage. Any conflicts will be adjudicated by third reviewer.

### Evidence Synthesis—Data Extraction and Meta-Analysis

We will pretested, piloted spreadsheets for data extraction and summarize characteristics of each study, including year and lead author; study population characteristics; the study interventions and controls in each study arm, which outcome were reported in the study; and risk of bias. If feasible, pooled estimates using both fixed-effects and random-effects models will be conducted, favoring random effects models unless inspection of the Forest plot of an outcome suggests that there is a risk of small studies effects, in which case fixed-effects may be used.^12^ For dichotomous outcomes, the relative risk, odds ratios, and absolute risk differences, with associated 95% CIs will be calculated. For continuous variables, mean differences or standardized mean differences with associated 95% CIs will be used. Narrative summaries of the data will be used when between-study differences or reported study data are not amenable to quantitative meta-analysis.^13^

### Evidence Appraisal

We will assess each trial using the Cochrane Risk of Bias 2 tool.^14^ To assess the overall certainty of effects, we will use the GRADE approach, rating the certainty of evidence for each outcome as high, moderate, low, or very low. The GRADE approach considers study risk of bias, inconsistency, indirectness, imprecision of estimates, and risk of publication bias to assess the overall level of certainty for each outcome.^11,15-18^ GRADE's standardized informative wording will be used to describe the summarized effects.^19^

### Formulation of Recommendations

The GRADE evidence-to-decision framework will be used by each PICO working to determine the strength and direction of each recommendation and to provide transparent judgments about each factor that influenced the recommendation.^20^ This includes the balance of effects (both desirable and undesirable), trade-offs between options, resources required, as well as acceptability and feasibility of each potential option. The recommendations will clearly include a strength of recommendation (strong vs conditional) and direction (either for or against a course of action). As is typical in GRADE, strong recommendations will typically require moderate or high certainty evidence and a clear balance in favor of a course of action vs its alternatives. If the certainty of evidence is lower, or the trade-offs more balanced between effects, costs, acceptability, or feasibility, the more likely it is that a recommendation will be conditional.

### Consensus Statements and Algorithm Development

Draft recommendations will reviewed in Bethesda, Maryland, at an in-person panel meeting in May 2026. The recommendations developed by each PICO working group will be reviewed by the entire panel. Additional expert consensus statements, used alongside the formal evidence-based recommendations, may also be used to facilitate implementation and understanding of each recommendation. To be approved, 80% of panelists without a relevant COI will need to vote in favor of the consensus statement. The approved recommendations and consensus statements will be used to guide the development of an algorithm to rapidly restore readiness. The algorithm will require 80% agreement of participants without relevant COI for final approval.

### Incorporation of Artificial Intelligence

Artificial Intelligence will be used to facilitate the literature searches, writing of evidence summaries, and the manuscript, with human oversight, reviewing, and editing of all materials used and produced. Elicit (https://elicit.com) will be used to supplement the traditional librarian-based literature searches; Open AI's GPT-4 will be used to draft sections of the manuscript before panelist review, editing, and approval.

## CONCLUSIONS

A rigorous systematic review using GRADE methodology, supplemented by an expert consensus process will be used to develop recommendations to enhance rapid restoration of cognitive readiness in military duty, sports, and other time-constrained and resource-constrained settings.

## COMMENTS

The authors publish a Methods paper describing a protocol for an upcoming project “Development of Guidelines on Rapid Restoration of Cognitive Readiness”, which includes possible pharmaceutical and nonpharmaceutical interventions to ensure unimpaired cognitive readiness and attention following concussion or subconcussive injury, insufficient sleep, fatigue, or any other disruption or deviation of an individual's neurocognitive baseline. Brain Trauma Foundation investigators will focus on health practices such as nutrition, physical conditioning, and rest, as well as pharmaceutical and supplement use to create guidelines to meet the urgent need for evidence-based restorative interventions. Outcomes of interest include: time to return to combat/sports, measures of performance in the field, injury rate, measures of readiness/attention/cognition, presence of symptoms (with particular focus on vestibular/ocular/motor screening), and any detrimental long-term effects of the interventions which ultimately are described.

Restoration of cognitive readiness is an important topic to examine, especially in an age with new heightened awareness of concussions and their long-term effects and awareness of the heightened risks of returning to activity too soon in stakeholder groups including far-forward military members, athletes in a variety of sports and echelons, garrison units, military and paramilitary units undergoing training, and at clinical facilities, where the results will have broad applications. Due to this likely broad applicability, secular interest, and potential financial and special interest entanglements associated with return to activities following (mild) traumatic brain injury (ie, concussion), such a project is inherently prone to bias and external influence. Prepublishing methods is an especially powerful tool for reducing bias in this case.

Notably, the authors prespecify that they will use artificial intelligence (AI)—including Ought's Elicit and Open AI's GPT-4 for help with literature searches, writing evidence summaries, and drafting sections of the manuscript before human panelist review, final draft creation, and publication. This AI use disclosure is consistent with Committee on Publication Ethics (COPE) recommendations, endorsed by Neurosurgery Publications, to indicate specific uses of AI in manuscript composition, while acknowledging that AI, particularly large language models, has become an increasingly-powerful tool for facilitating low-complexity but otherwise time-consuming research and publication-related tasks.

**Christopher Carr**

*Augusta, Georgia, USA*
